# A qualitative study of factors promoting EBM learning among medical students in Japan

**DOI:** 10.5116/ijme.62eb.7c19

**Published:** 2022-08-26

**Authors:** Yoshihiro Kataoka, Takami Maeno, Takashi Inaba, Sayaka Ninn, Masatsune Suzuki, Tetsuhiro Maeno

**Affiliations:** 1 Department of Primary Care and Medical Education, Faculty of Medicine, University of Tsukuba, Japan

**Keywords:** Active learning, evidence-based medicine, patient communication, qualitative research, undergraduate education

## Abstract

**Objectives:**

To identify the elements needed to facilitate undergraduate EBM learning among Japanese medical students.

**Methods:**

We conducted a qualitative study based on individual semi-structured interviews. Participants were physicians working at universities, teaching hospitals, or clinics who teach EBM to medical students. Purposive sampling was used to recruit participants via email through the researchers' acquaintances. Six physicians agreed to participate in the study and were interviewed individually from October 2019 to January 2020. The interviewees were asked about their own EBM learning and teaching experiences, what they kept in mind when teaching EBM to medical students, and what they felt was needed to improve current undergraduate EBM education. Interviews were recorded. Transcripts were analysed using thematic analysis.

**Results:**

Thematic analysis extracted five themes: finding foreground questions, observing role models, active learning, understanding patient backgrounds, and understanding the reason for learning EBM. To promote EBM education for medical students, it is first necessary for students to actively participate in clinical practice and identify foreground questions by observing their supervisors practicing EBM. In addition to acquiring skills in information retrieval and critical appraisal, understanding a patient's background leads to understanding the significance of learning EBM, which improves students' motivation to learn EBM.

**Conclusions:**

This study identified five themes that promote undergraduate EBM education. Curriculum development incorporating these elements would improve EBM education in Japan and other countries.

## Introduction

Evidence-based medicine (EBM) has become prevalent as "the conscientious, explicit, and judicious use of current best evidence in making decisions about the care of individual patients," since its introduction by Sackett et al. in the 1990s.^1^ The principles of EBM include asking appropriate clinical questions about patients, interventions, comparisons, and outcomes (PICO; step 1); searching for related and relevant evidence (step 2); critically appraising the evidence (step 3); integrating the evidence with clinical expertise and with patient's unique biology, values, and circumstances (step 4); and evaluating the effectiveness and efficiency in execution of steps 1-4 (step 5).^2^

For various professionals and disciplines, EBM has become an important process in medical decision-making.^3^ EBM is considered one of the most important aspects of undergraduate medical education.^4^^,^^5^ A number of studies have reported on undergraduate EBM curricula. The educational environment typically comprises classroom instruction, clinical settings (e.g., bedside rounds), and online learning.^6^ The timing of education can be preclinical, clinical, or scattered over multiple grades as a longitudinal curriculum.^6^ In terms of educational content, some curricula include more than one of the five principles of EBM, but few include step 1 (asking questions) or step 5 (reflection on steps 1-4).^7^ Although there have been many reports on the practice of EBM education, there are currently no established standards for when, how to educate, or how to evaluate the education.^7^^, ^^8^ So far, no specific curriculum was clearly found to be superior to others.^9^

When developing a medical education curriculum, it is essential to identify specific challenges in learning, learner needs, and learning objectives,^10^ and the same is true for EBM education. Given that medical education systems vary from country to country and differ in learning challenges and learning needs, it is important to establish an EBM curriculum that suits the actual educational situation in each country. Thus, it is necessary to first understand the learning environment for EBM education in individual countries.^11^

In Japan, a survey on the status of EBM education was conducted in 2001;^12^^, ^^13^ however, there have been no comprehensive reports since then. Further, while there is a four-week EBM elective training course at a single university^14^ and a year-long EBM education course for those who wish to attend,^15^ these programs have not undergone any standardized evaluation. Thus, the specific challenges in learning, learner needs, and learning objectives required to build an EBM curriculum suitable for the realities of undergraduate education in Japan remain to be determined.

Despite the fact that acquiring EBM techniques forms one of the learning goals of the model core curriculum for undergraduate medical education,^16^ few residents reportedly use EBM skills on a daily basis.^17^ It is possible that medical students do not fully understand the needs and objectives of EBM education. Thus, to determine the elements needed to engage medical students in EBM learning, it may be more effective, rather than focusing on the perceived needs of medical students as learners, to identify educator-perceived normative needs and learning goals.^18^

We performed a qualitative study to identify factors that would facilitate learning of EBM by medical students through personal interviews with educators.

## Methods

### Study design and participants

This qualitative study is based on individual semi-structured interviews. Based on the content of the interviews, we inductively derived factors that promote EBM learning among medical students.

Participants were recruited by purposive sampling. They were university faculty and physicians in teaching hospitals or clinics who were involved in EBM education. Participants were recruited so that all types of facilities (universities, hospitals, and clinics) were included. The sample was also collected so that age, gender, and years of post-graduation were varied. Study participants were recruited via individualized emails to potential participants who had experience with EBM education. If a potential participant refused to participate, the researchers asked the potential participant to refer their acquaintances for study participation.

### Interview and data collection method

Semi-structured interviews were conducted with each participant individually by YK between October 2019 and January 2020. Interviews were conducted using an interview guide developed by YK and TM. The interview questions mainly inquired about the contents of EBM education and what the interviewee keeps in mind when teaching EBM to medical students. The interviews were conducted in a private room at the participant's place of employment, such as a university or hospital. Each interview was recorded after obtaining consent from the participant. Written notes were also taken during the interview. Each interview lasted 40-60 minutes.

Each participant was asked about the following: 1) age, gender, number of years post-graduation, area of expertise, and number of years of experience in EBM education; 2) how and when they learned about EBM; 3) the curriculum and programs for EBM education at their institution; 4) their role in EBM education; 5) points they keep in mind when implementing EBM education; 6) their perceptions of medical students' competence in EBM; and 7) thoughts on what is needed to improve undergraduate EBM education.

### Data analysis

The data obtained in this study were analyzed using inductive thematic analysis.19, 20 A verbatim record of each interview was made by transcribing the recorded audio data. The participants checked the verbatim transcripts for errors to enhance trustworthiness. Two researchers (YK and TM) analyzed most of the text data. YK first performed coding of the data. TM and other authors then confirmed the contents. The themes were extracted inductively after the data were obtained. The coding process was carried out each time an interview was conducted. Specific suggestions for EBM education strategies obtained from the interviews were listed separately from the themes.

### Ethical issues

This study was conducted after obtaining approval from the ethics committee of the Faculty of Medicine, University of Tsukuba. Participants were informed that they were free to decide whether or not to cooperate in the study and that there were no disadvantages to not cooperating. In addition, they could choose to stop the interview at any point if they changed their minds about participating. There were no conflicts of interest between the interviewees and the researchers. After the interviews had been transcribed, the interviewees were asked to review the contents and delete any information that they did not want to be used in the analysis.

## Results

Six of the seven invited participants (two university faculty members and four teaching hospital or clinic physicians) gave consent to be interviewed. All interviewees were male, ranged in age from 30 to 58 years, graduated from medical school 7 to 34 years ago, and had 2 to 26 years of experience in EBM education.

Thematic analysis extracted five themes: finding foreground questions, observing role models, active learning, understanding patient backgrounds, and understanding the reason for learning EBM. We detail the five identified themes below, providing supporting quotes from the interviewees with information about their postgraduate year (PGY) and role (faculty member or teaching physician). We have added supplemental text to the quotes in brackets when further context was needed to facilitate understanding. In addition, we have also detailed some of the interviewees' specific suggestions for EBM education strategies.

### Finding foreground questions

When one interviewee learned about the existence of EBM, vague questions in daily practice emerged as "clinical questions," which inspired him to learn more about EBM.

"[In daily practice,] I wasn't registering my questions in the form of 'I don't understand this.' I had a lot of vague questions, and when I read Sackett's book, I was able to recognize that these were concrete questions." (Interviewee 1, PGY 34, teaching physician)

"I didn't understand that I didn't understand. I thought, 'Well, this might be interesting.'" (Interviewee 1, PGY 34, teaching physician)

These statements suggest that the interviewee's act of questioning clinical decisions that he had considered to be obvious promoted his EBM learning. The interviewee noted that the first step to practicing EBM is to realize the things of which he had previously been unaware.

However, medical students have little clinical experience and little general knowledge of diseases and syndromes, making it difficult for them to generate foreground questions.

"I think most of what students learn in class are background questions, and that knowledge is what they learn. Foreground questions are the ones that come up in clinical practice, or rather, the ones that develop in the field of clinical practice, so students who don't participate in clinical practice probably won't develop them at all." (Interviewee 5, PGY 7, faculty member)

### Observing role models

The interviewees suggested that for medical students, who were less likely to be aware of foreground questions, observing their supervisors practicing EBM could help them learn how EBM methods could be useful in a clinical setting.

"Personally, I think junior doctors would be inspired to see senior doctors enjoying learning and sharing the excitement of learning." (Interviewee 4, PGY 13, teaching physician)

"I think it's important to have the attitude that there are things we [supervising physicians] don't understand and that we're doing our best to research our questions on a daily basis." (Interviewee 4, PGY 13, teaching physician)

Additionally, the interviewees suggested that it was important for supervising physicians to be role models for medical students and to practice EBM on a regular basis. Seeing physicians practice EBM will motivate medical students to use EBM in a similar way.

"I think that the person teaching it should do [EBM] right, first of all. If you're in clinical practice, then you should practice EBM." (Interviewee 1, PGY 34, teaching physician)

"But I think the number of doctors [practicing the five steps of EBM] has increased tremendously compared to the past." (Interviewee 1, PGY 34, teaching physician)

### Active learning

Some interviewees thought that medical students needed to learn to become aware of their desire to help patients and their role within the medical team to enable them to actively learn EBM.

"In short, senior residents have to explain the patients' conditions to the patients and perform a variety of procedures, and even if they wanted to, they would not have enough time to find and read papers. Junior residents and students can help with that part of the work. That's the kind of role we wanted them to play, and we wanted them to work as a team." (Interviewee 2, PGY 31, faculty member)

One interviewee stated that he would sometimes try to stimulate passive students to make them aware of their role. Awareness of roles can motivate students to learn.

"I never treat them as students. It's not like, 'You're a student, so you'll have to watch.' In the mornings, I would deliberately ask, 'How is the patient who was admitted to hospital yesterday?' That means they would need to see the patient before rounds. That's what I try to get students to do. That's why they need to know their patients." (Interviewee 3, PGY 16, teaching physician)

The interviewees suggested that having a patient-focused perspective would make medical students more willing to learn apparently difficult skills, such as information seeking.

"The students learn that the process of searching for articles on clinical questions and applying them to their own patients is useful. This is the so-called 'aha moment,' and it is why the hurdle of searching for articles naturally becomes lower." (Interviewee 3, PGY 16, teaching physician)

### Understanding patient backgrounds

The interviewees suggested that it is important to communicate with each patient and to learn and understand their backgrounds and values. Several suggested that trying to understand the patient will motivate EBM education and practice. Rather than suddenly focusing on reading articles, students need to realize that the patient is the starting point for EBM.

"It's not just about focusing on what is the outcome. It's about knowing the person. When thinking of the patients themselves, the outcome comes naturally." (Interviewee 4, PGY 13, teaching physician)

"For me personally, when I think about PICO as the first step, information about the outcome is extremely important, so I want students to talk with patients very carefully and explore their values." (Interviewee 4, PGY 13, teaching physician)

### Understanding the reason for learning EBM

Remarks from the interviewees suggested that medical students should realize why they learn EBM through communication with patients. This might be more important than mastering EBM skills such as information retrieval and critical appraisal of articles. Understanding that EBM is used for the benefit of the patient is important for medical students to learn EBM.

"At the time, I didn't understand the necessity of learning EBM, which is something I understand in hindsight; I don't really feel that I should have learned about EBM techniques first." (Interviewee 5, PGY 7, faculty member)

"Personally, I don't think students need to learn about critical appraisal at all. I think critical appraisal is an art, to a certain extent."(Interviewee 4, PGY 13, teaching physician)

One interviewee suggested that understanding the rationale for PICO, which is to understand patient backgrounds, should be an important theme.

"Formulation of questions with PICO is only a tool. The purpose is to be curious and learn about patient backgrounds. In that sense, I would like to introduce this tool to students at the first step of EBM." (Interviewee 4, PGY 13, teaching physician)

**Figure 1:**
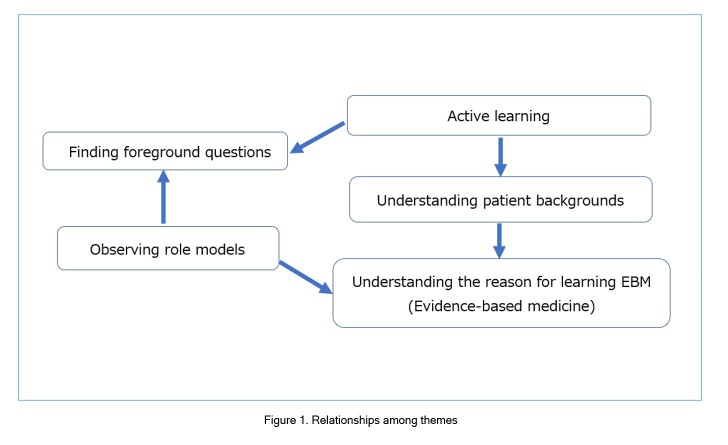
Relationships among themes

Figure 1 shows the relationships among the themes. First, medical students observe supervising physicians practicing EBM. This experience promotes their awareness of foreground questions in clinical practice and awareness of reasons for using EBM as a method of decision making. When students become aware of their roles and are learning actively, they find more foreground questions and understand patients background deeper. These experiences are not directly related to specific techniques of EBM such as information seeking or critical appraisal, but they deepen an understanding of why EBM is used in clinical practice and gain motivation for learning EBM.

### Suggestions of specific strategies to help medical students learn EBM

During the interviews, the participants suggested specific strategies to help medical students learn EBM. One was to use the study of statistics and history of medicine during the preclinical phase to help medical students realize the impact of medical articles on clinical decisions. Another was to incorporate EBM principles as additional learning in their areas of interest or an area in which the students feel they have a strong understanding once they have acquired some background knowledge in each clinical discipline. The interviewees also suggested that students could divide their role into two, as a doctor and as a patient, and explain the research-proven effects of a drug to patients as a way to learn how to apply evidence in practice. Finally, rather than educating students about EBM in a specific discipline, some interviewees suggested creating a curriculum that allows them to learn EBM across disciplines.

## Discussion

The analysis of the interviews revealed that students' awareness of their roles and active learning can facilitate learning of EBM by leading to understanding of patient backgrounds and discovery of foreground questions. In detail, while becoming aware of foreground questions in clinical practice is a trigger for practicing and learning EBM, medical students with little clinical experience are first motivated by observing their supervisor, a role model, practice EBM. As medical students begin to realize their role in the care of patients, they will understand the significance of the EBM methodology, which is essentially to solve problems for patients. In learning EBM, it is important to first understand patient backgrounds and values to enable the formulation of questions through PICO. An appropriate learning goal for students is to understand why EBM is practiced rather than how to practice it.

Although there are signs of gradual change in Japan, clinical practice is still often an observation-based learning method.^21^ By giving medical students specific roles in clinical practice, their learning changes from observational to participatory and they become able to proactively think about patients' health issues.^21^ Proactively interacting with patients enables students to think about the patient's highest priorities and what it means to be healthy, which are the true outcomes for the patient, leading to the generation of foreground questions based on PICO.

To encourage medical students to formulate their own clinical questions, application of legitimate peripheral participation^22^ may be useful. Legitimate peripheral participation is a process in which the learner becomes a legitimate member of a workplace and learns from the periphery by gradually deepening their degree of participation by emulating the person at the center of the workplace. By creating a learning environment in which medical students participate in ward teams or outpatient clinics as legitimate members, they begin to acquire knowledge and skills in a step-by-step manner by observing how doctors practice EBM, even if they are unable to immediately learn EBM practices on the job. This will require a change in faculty awareness and curricula that welcome medical students as part of the team. This strategy can be applied to EBM education in countries where, like Japan, participation by medical students is challenging.^23^

Based on the remarks of educators in the interviews, it may be more important for medical students to learn why EBM is necessary for clinical practice than to learn the specific skills and knowledge content of EBM.

Although EBM education for medical professionals often focuses on skills related to step 3,^24^ the results of this study suggest that we should rather focus on the "why" of learning EBM. Thus, it may be better for early-career medical students to begin learning EBM from step 4, understanding that step 1 is the starting point, and then that information retrieval skills (step 2) and critical appraisal (step 3) are methods for obtaining answers to the questions raised in step 1. Learning EBM in this order will increase students' internal motivation for learning and help them refine their EBM methods once they have graduated as physicians.

One interviewee suggested that preclinical students could learn EBM by thinking about how medical articles affect clinical practice through the study of statistics. How early-career medical students can effectively learn EBM without abundant medical knowledge is a major consideration when establishing an EBM curriculum.^25^ One report lists "understanding of basic principles and simple examples of medical decision making" and "the scientific method applied to the study of medical science" as items that students should learn in the first 2 years of medical school.^26^ Meanwhile, exercises such as scenario-based clinical questioning during later years of medical school were recommended. This gradual progression of learning from lower to higher grades^25^ could facilitate medical students' understanding of their need to learn EBM while increasing their prerequisite background knowledge for EBM.

This study gained insights from interviews with supervisors about ways to facilitate EBM learning among medical students. The ideas are compelling because they are derived from the supervisors' experiences with developing their own knowledge and skills in EBM and current teaching.

There are several limitations to this study. One is that the interviewees were educators rather than learners. If students had been interviewed instead, we might have been able to identify factors that promote EBM learning from the students' perspective. However, we interviewed educators to obtain their views on challenges associated with facilitating EBM learning as well as solutions to those challenges. Second, the number of participants (n=6) was small. In thematic analysis, the number of samples needed for all codes to be extracted depends on the characteristics of the subject and the research theme, but for a relatively homogeneous population, a sample of approximately 12 people is needed.^27^ However, the number of supervisors who teach EBM in the field in undergraduate medical education in Japan is limited;^13^ thus, we were unable to recruit more participants. We recruited male and female participants, but it was difficult to find female participants. This is probably because the percentage of female faculty members in Japanese medical schools is as low as <30%.^28^ If we can increase the number of subjects and include women in the survey in the future, we will be able to deepen our findings.

## Conclusions

To promote undergraduate EBM learning, medical students first need to observe role models and actively participate in finding foreground questions and understanding patient backgrounds. Understanding the significance of EBM will facilitate EBM learning rather than individual EBM techniques. Based on the findings of this study, continuous and systematic incorporation of EBM into clinical education will improve the curriculum of undergraduate EBM learning in Japan and other countries, especially those with inadequate clinical participation by medical students. Future studies asking medical students about their readiness to learn EBM and past experiences with it will strengthen our findings.

### Acknowledgments

The authors would like to thank all participants for cooperating in the interview.

### Conflict of Interest

The authors declare that they have no conflict of interest.
